# Effectiveness and safety of Yufeng Ningxin for the treatment of essential hypertension: A protocol for systematic review and meta-analysis

**DOI:** 10.1097/MD.0000000000024858

**Published:** 2021-03-05

**Authors:** Mingyan Huang, Linzi Long, Mi Deng, Zikai Yu, Hua Qu, Ling Tan, Yuxuan Peng, Changgeng Fu

**Affiliations:** a Xiyuan Hospital of China Academy of Chinese Medical Sciences; b Beijing University of Chinese Medicine, Beijing; c Fujian University of Traditional Chinese Medicine, Fuzhou; d National Clinical Research Center for Cardiovascular Diseases of Traditional Chinese Medicine, Beijing, China.

**Keywords:** essential hypertension, meta-analysis, protocol, systematic review, Yufeng Ningxin

## Abstract

**Background:**

Essential hypertension is the primary cause of death and disability and it has become a major public health problem globally. Yufeng Ningxin (YFNX) is a commonly used Chinese patent medicine in treating essential hypertension. The objective of this protocol is to evaluate the effectiveness and safety of YFNX for the treatment of essential hypertension.

**Methods:**

Randomized controlled trials (RCTs) in relation to the effectiveness and safety of YFNX in the treatment of essential hypertension will be systematically searched and collected from the following databases: PubMed, EMBASE, Cochrane Library, Chinese National Knowledge Infrastructure, Wanfang Database, Chinese Biomedical Literature Database, and Chinese Scientific Journal Database from the database inception to January 1, 2021. The data screening and extraction will be carried out by 2 different reviewers. The quality of randomized controlled trials will be assessed based on the version 2 of the risk-of-bias tool for randomized trials (RoB 2) in the Cochrane Handbook. The reduction of systolic blood pressure (SBP) and diastolic blood pressure (DBP) will be served as the primary outcome. The secondary outcomes will include average SBP and average DBP during the day and the night measured by 24 hours ambulatory blood pressure monitoring, the clinical effectiveness rate, scores of traditional Chinese medicine syndrome, clinical symptoms, the quality of life and adverse events. Statistical analysis will be conducted with Review Manager 5.3 and STATA 14.0 software.

**Conclusion:**

This systematic review will provide strong evidence to assess the effectiveness and safety of YFNX in the treatment of essential hypertension.

**Trial registration number:**

INPLASY202110059.

## 1 Introduction

Essential hypertension is the primary risk factor for deadly cardio-cerebrovascular diseases such as strokes, heart failure, myocardial infarction, atrial fibrillation, etc. As a result, hypertension is a major cause of death in the world, inducing 10.4 million deaths every year.^[1]^ For hypertensive patients, the purpose of antihypertensive treatment is to effectively prevent or delay the occurrence of cardio-cerebrovascular diseases. Lowering blood pressure (BP) can significantly reduce the premature morbidity and mortality of cardio-cerebrovascular complications.^[2]^ Common antihypertensive methods mainly adopt the lifestyle improvement and drug treatment such as calcium channel blocker, angiotensin converting enzyme inhibitor, angiotensin receptor blocker, β-blockers, diuretics, etc. Despite the adoption of multiple treatment measures, the prevalence of hypertension continues to increase, leading to an increasing trend in the incidence and mortality of cardio-cerebrovascular diseases worldwide.^[3]^ The number of patients with hypertension will increase by 15% to 20% and will approach 1.5 billion by 2025.^[4]^ As a result, hypertension is a major global public health problem.

According to the 2012–2015 China Hypertension Survey, the prevalence of hypertension in China is still on the rise and it has increased from 18% to 23.2% compared with the survey results in 2002.^[5]^ Although the awareness rate, treatment rate, and control rate of hypertension have increased significantly, they are still at a low level. Among Chinese adults over 18 years old, 244.5 million people suffer from hypertension. Another 41.3% (453 million) people are under the prehypertension stage. Among hypertensive patients, 46.9% patients knew their condition, 40.7% patients received prescribed antihypertensive medications, but only 15.3% patients had their BP under control.^[6]^ In addition, conventional antihypertensive medicine can cause headache, cough, diarrhea, angioneurotic edema, and other adverse reactions, which affects the medication compliance of patients.^[7]^ Therefore, seeking new complementary and alternative therapies is of great significance for the prevention and treatment of hypertension. An evidence map involving 5920 randomized controlled studies, 16 guidelines, 139 system reviews, expert consensus, and clinical pathways showed that traditional Chinese medicine can lower BP, reduce target organ damage and improve major clinical symptoms such as dizziness and headache. In conclusion, combination of Chinese and Western medicine can enhance the effect of lowering BP and reduce adverse reactions.^[8]^

Yufeng Ningxin (YFNX), extracted and processed from the Pueraria lobata, is a commonly used Chinese patent medicine for the treatment of hypertension. It has the function of resolving tetany and relieving pain, enhancing brain and coronary blood flow as well as lowering BP. It is included in the 2020 edition of the Pharmacopoeia of the People's Republic of China.^[9]^ High performance liquid chromatography analysis shows that the main active ingredient of YFNX is puerarin, and its content shall not be lower than the specified 13 mg/tablet.^[10,11]^ Puerarin is an isoflavone compound that has strong activity in the cardiovascular system. It can promote vasodilation, protect endothelium, improve insulin resistance, lower BP, and inhibit vascular remodeling.^[12–15]^ Clinical studies have shown that YFNX can significantly lower BP and improve hypertension symptoms such as dizziness, headache, etc.^[16–18]^ However, there is still no strong evidence showing the effectiveness and safety of YFNX in treating hypertension. Therefore, this study will conduct a high-quality systematic review and meta-analysis on the effectiveness and safety of YFNX in the treatment of essential hypertension so as to provide reference for clinical applications.

## 2 Methods

### 2.1 Study registration

The protocol has been registered in INPLASY (registration number: INPLASY202110059). If there are any adjustments in this study, we intend to revise and update them in the final publication. This review will be conducted and reported strictly according to the Preferred Reporting Items for Systematic Reviews and Meta-Analysis (PRISMA) Statement.^[19]^

### 2.2 Eligible criteria for study selection

#### 2.2.1 Types of studies

We will include all randomized controlled trials (RCTs) of YFNX in the treatment of essential hypertension, regardless of language, and publication types.

#### 2.2.2 Types of participants

The participants included must have a diagnosis of essential hypertension. The diagnosis of essential hypertension will refer to the criteria published in “2020 International Society of Hypertension global hypertension practice guidelines,”^[3]^ which is defined as systolic blood pressure (SBP) ≥140 mm Hg and/or diastolic blood pressure (DBP) ≥90 mm Hg. We will not consider the patient's age, gender, ethnicity, and region.

#### 2.2.3 Types of interventions

Participants in the intervention group accepted the treatment of YFNX, and the control group accepted YFNX placebo or recommended antihypertensive agents such as calcium channel blocker, angiotensin converting enzyme inhibitor, angiotensin receptor blocker, diuretics, beta-adrenergic blocking agents, etc. If the intervention group took YFNX combined with recommended antihypertensive agents, the same antihypertensive agents must be used in the control group. The various dosage forms of YFNX will include dripping pills, tablets, capsules, granules, etc. Its dosage and duration will be unlimited. The study using other Chinese medicines will be excluded.

#### 2.2.4 Types of outcome measures

##### 2.2.4.1 Primary outcomes

The primary outcomes will be the reduction of SBP and DBP.

##### 2.2.4.2 Secondary outcomes

The secondary outcomes will include average SBP and average DBP during the day and the night measured by 24 hours ambulatory blood pressure monitoring, the clinical effectiveness rate, scores of TCM syndrome, clinical symptoms, the quality of life, and adverse events.

### 2.3 Search methods for the identification of studies

#### 2.3.1 Electronic searches

We plan to conduct a systematic search of studies through the following databases: PubMed, EMBASE, Cochrane Library, Chinese National Knowledge Infrastructure, Wanfang Database, Chinese Biomedical Literature Database, and Chinese Scientific Journal Database from the start date of the databases to January 1, 2021. The retrieval terms include YFNX, hypertension and RCTs. The detailed retrieval strategy for PubMed is shown in Table 1, and other electronic databases will also be searched similar to the retrieval strategy of PubMed.

**Table 1:** Retrieval strategy for PubMed.

#1	Hypertension [Title/Abstract]
#2	high blood pressure [Title/Abstract]
#3	Blood Pressure, High [Title/Abstract]
#4	essential hypertension [Title/Abstract]
#5	primary hypertension [Title/Abstract]
#6	gao xue ya [Title/Abstract]
#7	#1 or #2 or #3 or #4 or #5 or #6
#8	Yufengningxin [Title/Abstract]
#9	Yufeng ingxin [Title/Abstract]
#10	YFNX [Title/Abstract]
#11	#8 or #9 or #10
#12	randomized controlled trial [All Fields]
#13	controlled clinical trial [All Fields]
#14	randomized [All Fields]
#15	#12 or #13 or #14
#16	#7 and #11 and #15

#### 2.3.2 Search for other resources

We will also search grey documents and clinical trial registers including the Chinese Clinical Trial Register, WHO International Clinical Trials Registry Platform, and the Clinical Trials, to supplement the electronic databases.

### 2.4 Data collection and analysis

#### 2.4.1 Selection of studies

**Figure 1:**
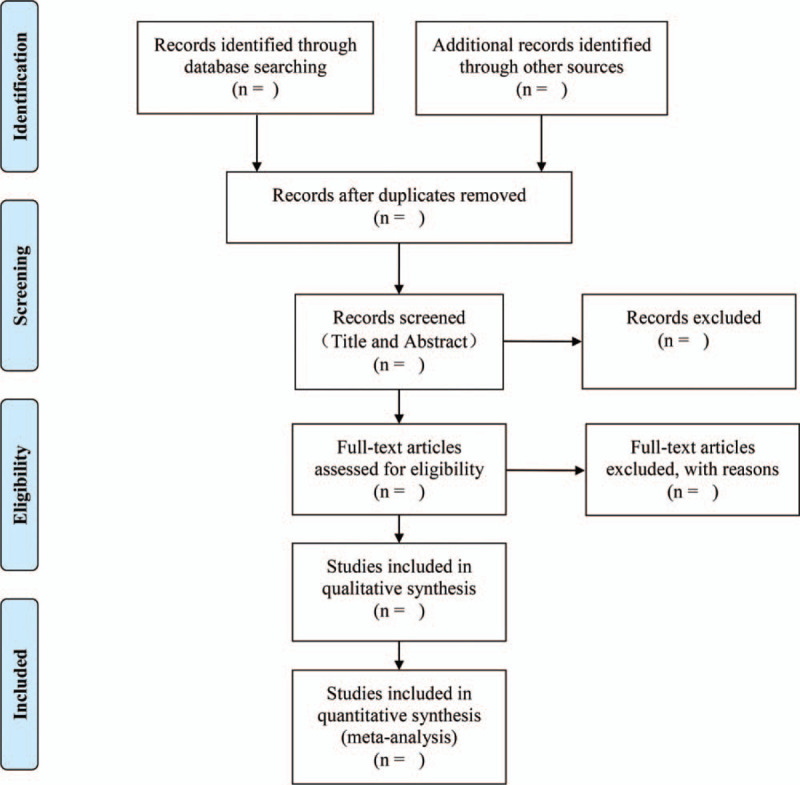
Flow chart of preferred reporting items for systematic review and meta-analysis (PRISMA).

All retrieved literature will be imported into Endnote X9 software. Two authors (Mingyan Huang and Linzi Long) will independently sift the potentially eligible literature by screening the titles and abstracts on the basis of the inclusion criteria. After removing uncorrelated and duplicated research, they will further look through the full text to evaluate their eligibility. Then, all studies selected by the authors will be cross-checked, and if there are any disagreements, they will be discussed and resolved by a third author (Changgeng Fu). The literature screening and selection processes will be performed according to the PRISMA flow chart shown in Figure 1.

#### 2.4.2 Data extraction and management

Two authors (Mingyan Huang and Linzi Long) will independently extract the data from the last included studies according to the predefined data collection table. The table will incorporate study characteristics (the first author, country, title, journal, publication year, etc.), study design information (randomization, blinding, allocation hiding, selective reporting, loss to follow-up, and any other bias information), subjects’ information (number of subjects, age, sex ratio, diagnostic criteria, and accompanying disease severity), intervention and comparison characteristics (dose, dosage form, frequency, and duration), and outcomes (primary outcomes and secondary outcomes). Any disagreements will be worked out through discussion with a third researcher (Changgeng Fu). If there is any unclear or missing information, we are going to consult the author of the original study by email or telephone. All extracted data will be imported into RevMan 5.3 software.

#### 2.4.3 Assessment of risk of bias in included studies

The version 2 of the risk-of-bias tool for randomized trials (RoB 2) is provided by Cochrane Handbook for Systematic Reviews of Intervention version 6.1 (updated September 2020).^[20]^ RoB 2 will be used to evaluate the risk of bias in each included study by 2 independent authors. The main items include randomization bias (random sequence allocation, allocation concealment), intervention bias, measurement bias, missing outcome data bias, and reporting bias. According to the overall risk-of-bias judgment, each bias is divided into 3 levels, including low risk, high risk, and some concerns. If there are any differences in the evaluation, we will discuss and negotiate with the third author (Changgeng Fu) to reach a consensus.

#### 2.4.4 Measures of treatment effect

We will use the rate ratio and the 95% confidence interval to analyze the dichotomous data. For the continuous data, the mean difference and 95% confidence interval will be used to show the results between the groups.

#### 2.4.5 Dealing with missing data

As for the missing or unclear data in the included literature, the reviewers will consult the author to obtain the missing or unclear information by email, telephone or other means. If no more information is available, we will only analyze the accessible data. Meanwhile, we will also emphasize the potential impact of the missing data.

#### 2.4.6 Assessment of heterogeneity

Before data synthesis, clinical and methodological heterogeneity will be totally analyzed and discussed. The *I*^2^ test will be used to assess the statistical heterogeneity in the included studies. If *I*^2^ is ≤50%, that means there is no significant heterogeneity among studies. Conversely, it means that there is significant heterogeneity.

#### 2.4.7 Data synthesis and analysis

We will utilize the RevMan 5.3 software (Cochrane Collaboration, Copenhagen, Denmark) to perform data analysis. When *I*^2^ ≤ 50%, the fixed effect model will be used for meta-analysis. Otherwise, when *I*^2^ > 50%, the random effect model will be selected to evaluate the outcome data. When significant heterogeneity is observed, we are going to conduct subgroup analysis, sensitivity analysis, or descriptive analysis to analyze the potential causes for heterogeneity. If the quantitative synthesis is not suitable, we will conduct a descriptive analysis.

#### 2.4.8 Publication bias

The funnel plot analysis will be conducted to evaluate publication bias when there are more than 10 trials in the study. If there are fewer than 10 trials in the study, we will conduct a quantitative analysis through the Egger test by the means of the STATA 14.0 software.

#### 2.4.9 Subgroup analysis

If the included studies have obvious heterogeneity and there are more than 10 trials, the subgroup analysis will be conducted on the basis of the age, sex, the severity of hypertension, and the dosage of YFNX.

#### 2.4.10 Sensitivity analysis

In order to ensure the accuracy of research conclusions, we will conduct a sensitivity analysis by eliminating each study individually or transforming the category of the effect model.

#### 2.4.11 Grading the quality of evidence

In the systematic review, the quality of evidence will be classified into 4 levels: “high quality,” “moderate quality,” “low quality,” and “extremely low quality” by the means of the GRADE tool.

#### 2.4.12 Ethics and dissemination

This article assembles published studies for data analysis rather than the individual privacy data of patients. So, there is no need to require ethical approval. The results of this review will be published in peer-reviewed journals and presented at relevant conferences.

## 3 Discussion

Hypertension is a leading cause of death and disability and it has become a major public health problem globally.^[21]^ YFNX has been widely used in the treatment of essential hypertension in China and it is effective components puerarin can protect endothelium,^[15]^ dilate blood vessels,^[22]^ improve insulin resistance,^[13]^ improve vascular remodeling, and end-organ damage.^[15]^ Therefore, it is necessary to conduct a systematic review and meta-analysis to verify the effectiveness and safety in treating essential hypertension. It is hoped that this research can find more rigorous medical evidence for YFNX in the treatment of essential hypertension, so as to provide reference for clinical practice. However, there are still some underlying limitations in this study. First of all, there is still a lack of high-quality, multicenter and large sample clinical trials, which will affect the authenticity of the evidence. Secondly, the different dosage forms and dosages of YFNX may cause significant heterogeneity in results. In consequence, high-quality, large-sample RCTs should be executed in the future to validate the clinical efficacy of YFNX.

## Author contributions

**Conceptualization:** Mingyan Huang.

**Data curation:** Linzi Long, Mi Deng.

**Formal analysis:** Hua Qu, Zikai Yu.

**Funding acquisition:** Changgeng Fu.

**Methodology:** Mingyan Huang, Ling Tan.

**Project administration:** Changgeng Fu.

**Resources:** Hua Qu, Yuxuan Peng.

**Software:** Zikai Yu, Hua Qu.

**Writing – original draft:** Mingyan Huang, Mi Deng.

**Writing – review & editing:** Mingyan Huang, Linzi Long.
